# Total hip replacement in young adults with hip dysplasia

**DOI:** 10.3109/17453674.2011.627495

**Published:** 2011-11-24

**Authors:** Hartofilakidis George, T. Nikolaos

**Affiliations:** University of AthensRoidis; 3rd Paedic Dept, KAT Hospital, Athens

*Sir—*It was with great interest that we read your article: “Total hip replacement in young adults with hip dysplasia: age at diagnosis, previous treatment, quality of life, and validation of diagnoses reported to the Norwegian Arthroplasty Register between 1987 and 2007” (Acta Orthop 2011; 82 (2): 149-54). We wish, however, to comment on the indiscriminate use of the term “dysplasia” for the whole spectrum of the deformity, which is not in agreement with the variety of underline pathology. In our opinion, in order to avoid confusion and diagnostic inaccuracies, the term ”dysplasia” should be reserved for the milder types of hip deformity.

It is clear, we still do not have an agreed terminology, covering the entire pathology of congenital deformities of the hip or a generally accepted classification that will improve our communication, our understanding of the natural history of the different types of the deformity, treatment planning and evaluation of results of various treatment.

We favor the use of the term ”Congenital Hip Disease”, for the whole spectrum of the deformity. Accordingly, it can be classified in infants as: dysplasia, subluxation, and dislocation (Wedge and Wasylenko 1979, Weinseine 1987), and in adults as: dysplasia, low dislocation and high dislocation (Hartofilakidis et al. 1988, 1996, 2000, Hartofilakidis and Karachalios 2000, Karachalios and Hartofilakidis 2010).

In one of our previous studies on the epidemiology, demographics, and natural history of congenital hip disease, based on 231 adult patients (356 hips), we confirmed that 170 hips (48%) were dysplastic, 85 (24%) had low dislocation, and 101 (28%) had high dislocation. 338 of the hips (95%) were in women. In the dysplastic hips, no history of hip disease in childhood was recorded. The disease had been undiagnosed until the onset of symptoms at a mean age of 35 (18–40) years. In patients with low and high dislocation, diagnosis was established since early childhood. Limping was present from infancy, with pain usually starting between the ages of 25 and 30.
