# Supplementary material for: Total hip replacement in young adults with hip dysplasia
Source: Acta Orthop. 2011 Nov 24;82(5):635–6. doi: 10.3109/17453674.2011.627495 (PMC3242965; doi:10.3109/17453674.2011.627495)
Supplement: Related texts

# Letter: Total hip replacement in young adults with hip dysplasia

**Published:** 2011-11-24

**Authors:** Ingvild Ø Engesæter

**Affiliations:** The Norwegian Arthroplasty Register, Department of Orthopedic Surgery, Haukeland University Hospital; Bergen, NorwayIngvild.Ovstebo.Engeseter@helse-bergen.no

*Sir—*First of all, we thank you for your interest in our article. The terminology of hip dysplasia is an essential topic and we agree on the importance of precise terms. The vocabulary has changed over time, and the wide spectre of severity of the condition and different ages at diagnosis may partly explain this conceptual dissatisfaction and confusion.

In your letter, you recommend the use of “Congenital Hip Disease” for the whole spectre of the deformity with a further sub-classification into 3 groups depending on the severity. You also refer to your interesting work on adult patients with osteoarthritis secondary to a congenital hip disease (Hartofilakidis et al. 2000). One of your findings is that patients with the mildest affection (dysplasia) had no history of hip disease in childhood. This supports the findings in our study (high age at diagnosis (median 7.8 years)), but also indicates that the term “congenital” can be misleading in this context. Americal Academy of Pediatrics, Academy of Orthopaedic Surgons (AAOS), and The Pediatric Orthopedic Society of North America (POSNA) have advocated the use of developmental dysplasia of the hip (DDH) as the terminology includes all cases that are clearly congenital and those that are developmental, incorporating subluxation, dislocation and dysplasia of the hip (Clinical practice guideline: early detection of developmental dysplasia of the hip. Committee on Quality Improvement, Subcommittee on Developmental Dysplasia of the Hip. American Academy of Pediatrics 2000). In our study we have used the DDH-term in combination with hip dysplasia, two terms also used interchangeably in the literature.

Your recommendation regarding the sub-classification of the condition is clearly useful. In our study, we had unfortunately no opportunity for such a classification, as the initial radiographs for a large proportion of the patients were missing. Another challenge would have been to define radiological measurements with corresponding cut-off values for the various subgroups.

## References

[CIT0001] (2000). Clinical practice guideline: early detection of developmental dysplasia of the hip. Committee on Quality Improvement, Subcommittee on Developmental Dysplasia of the Hip. American Academy of Pediatrics. Pediatrics.

[CIT0002] Engesæter IØ, Lehmann T, Laborie LB, Lie SA, Rosendahl K, Engesæter LB (2011). Total hip replacement in young adults with hip dysplasia: age at diagnosis, previous treatment, quality of life, and validation of diagnoses reported to the Norwegian Arthroplasty Register between 1987 and 2007. Acta Orthop.

[CIT0003] Hartofilakidis G, Karachalios T (2000). Total hip relplacement in congenital hip disease. Surgical techniques in orthopaedics and traumatology.

[CIT0004] Hartofilakidis G, Stamos K, Ioannidis TT (1988). Low friction arthroplasty for old untreated congenital dislocation of the hip. J Bone Joint Surg (Br).

[CIT0005] Hartofilakidis G, Stamos K, Karachalios T, Ioannidis TT, Zacharakis N (1996). Congenital hip disease in adults. Classification of acetabular deficiencies and operative treatment with acetabuloplasty combined with total hip arthroplasty. J Bone Joint Surg (Am).

[CIT0006] Hartofilakidis G, Karachalios T, Stamos K (2000). Epidemiology, demographics and natural history of congenital hip disease in adults. Orthopaedics.

[CIT0007] Karachalios T, Hartofilakidis G (2010). Aspects of current management. Congenitial hip disease in adults. J Bone Joint Surg (Br).

[CIT0008] Wedge JH, Wasylenko MJ (1979). The natural history of congenital disease of the hip. J Bone Joint Surg (Br).

[CIT0009] Weinseine SL (1987). Natural history of congenital hip dislocation and hip dysplasia. Clin Orthop.
